# Protective effect of honokiol on cadmium-induced liver injury in chickens

**DOI:** 10.1016/j.psj.2024.104066

**Published:** 2024-07-06

**Authors:** Xiaoqian Huang, Junzhao Yuan, Jianhong Gu, Yassar Abbas, Yan Yuan, Zongping Liu, Hui Zou, Jianchun Bian

**Affiliations:** ⁎ College of Veterinary Medicine, Yangzhou University, Yangzhou 225009, Jiangsu, China; † College of Veterinary Medicine, Henan University of Animal Husbandry and Economy, Zhengzhou 450000, Henan, China; ‡ Jiangsu Co-innovation Center for Prevention and Control of Important Animal Infectious Diseases and Zoonoses, Yangzhou 225009, Jiangsu, China; § Department of Animal Sciences, Jhang Campus, University of Veterinary and Animal Sciences, Jhang 54590, Pakistan

**Keywords:** cadmium, gap junctional, intercellular communication, honokiol, trace elements

## Abstract

Cadmium (**Cd**), a highly toxic heavy metal in the environment, poses a significant threat to livestock and poultry farming. Honokiol (**HNK**), a Chinese herbal extract with potent antioxidant activity, acts through oxidative damage and inflammation. Cd induces oxidative stress and causes liver damage in animals. However, whether HNK can alleviate Cd-induced liver injury in chickens and its mechanism remains unclear. In this study, the 48 chickens were randomly allocated into 4 groups, control group, Cd group (70 mg/kg Cd), HNK group (200 mg/kg HNK) and Cd + HNK group (70 mg/kg Cd+200 mg/kg HNK). Results showed that HNK improved the Cd induced reduction in chicken body weight, liver weight, and liver coefficient. HNK recovered the Cd induced liver damaged through increased serum liver biochemical indexes, impaired liver oxidase activity and the disordered the expression level of antioxidant genes. HNK alleviated Cd induced pathological and ultrastructure damage of liver tissue and liver cell that leads apoptosis. HNK decreased Cd contents in the liver, Cd induced disturbances in the levels of trace elements such as iron, copper, zinc, manganese, and selenium. HNK attenuated the damage to the gap junction structure of chicken liver cells caused by Cd and reduced the impairment of oxidase activity and the expression level of antioxidant genes induced by Cd. In conclusion, HNK presents essential preventive measures and a novel pharmacological potential therapy against Cd induced liver injury. Our experiments show that HNK can be used as a new green feed additive in the poultry industry, which provides a theoretical basis for HNK to deal with the pollution caused by Cd in the poultry industry.

## INTRODUCTION

Cadmium (**Cd**), a heavy metal element that is not needed by living organisms. Cd in the environment mainly comes from mining, automobile exhaust emissions, industrial manufacturing, and so on. The heavy metal Cd in the environment is eventually absorbed by crops and gradually enriched by livestock and poultry in the livestock industry, endangering human health and aggravating chronic diseases (Satarug et al., 2010; Wang et al., 2024). Cd can produce cytotoxicity, extensively bind to various proteins in the cell, destroy lysosomes, mitochondria, and endoplasmic reticulum biofilms, generate free radicals, cause oxidative stress damage to cells, and eventually induce apoptosis (Genchi et al., 2020; Zhang et al., 2024).

The traditional Chinese medicine honokiol (**HNK**) is mainly derived from the bark of the deciduous tree *Magnolia officinalis*, which is widely distributed throughout China (Dikalov et al., 2008). In the field of molecular biology, HNK has been proven to have anti-inflammatory, antibacterial, antiviral, antioxidant, and other biological effects (Wu et al., 2011; Kataoka et al., 2021). HNK, as a poly benzene ring compound, has been shown to have good antioxidant effects (Zhao and Liu, 2011). HNK has also been used to alleviate drug-induced hepatotoxicity, liver fibrosis, and diabetes in chicken (Ding et al., 2021; Kataoka et al., 2021). In cardiomyocytes, HNK has been shown to protect mitochondrial function, primarily by activating the mitochondrial SIRT3-SOD2 pathway (Pillai et al., 2015). In a word, HNK as a new type of green plant extract is gradually being used more and more. However, it was not known whether HNK could exert a protective effect against Cd-induced oxidative stress in chicken liver, so this study started the experiment mainly from the perspective of oxidative stress.

The antioxidant system is composed of multiple systems in tissue cells, including antioxidant enzymes such as CAT, T-SOD, GSH-PX, and antioxidant substances such as GSH and MDA, among which different types of trace elements play a major role in the biological activity of antioxidant proteins (Sun et al., 2023). The glutathione peroxidase family Gppoly benzene (including Gpx 1, Gpx 2, Gpx 4) and the thioredoxin family TrxR (including TrxR1, TrxR2, and TrxR3) are 2 selenoproteins that play an important role in cellular antioxidant function. When cells are in a state of stress, such as a lack of oxygen, high oxygen and ultraviolet radiation, chemicals, viruses, surgery, malnutrition. Heat shock proteins (**HSP**) are secreted by will heavily, including Hsp70 and Hsp90, Hsp60, Hsp27, Hsp40. It can form complexes with various proteins in the cell, and participate in protein folding, transport, activation, degradation, and protein signal transmission, which is also known as molecular chaperones (Malik and Lone, 2021; Zininga et al., 2018).

There is no doubt about the importance of the layer chicken industry in human lives. Eggs and chicken are indispensable in modern life. The impact of heavy metals in drinking water or feeding on the growth and development of laying chickens directly affects their production, and the heavy metals accumulated in the liver or chicken will eventually threaten human health. At present, there is still a lack of reasonable and effective drugs and methods to deal with heavy metal poisoning, but the method of adding natural plant extracts in feed is considered to be a reasonable and effective way to deal with heavy metal poisoning. HNK was first used in the treatment of heavy metal poisoning induced by Cd in chickens to find new green and natural drugs for the treatment of heavy metal poisoning.

## MATERIALS AND METHODS

### Animal Care and Experimental Design

All experimental procedures were conducted by the Guide for the Care and Use of Laboratory Animals of the National Research Council and were approved by the Animal Care and Use Committee of the Yangzhou University (Approval ID: 202103-348). A total of 48, 1-day-old Sanhuang chickens were purchased from Jiangsu Poultry Institute, all of which were female chicks for egg production. The chickens were placed in a clean animal room and immediately provided with clean drinking water. Three hours later, they were given pellet feed. The raw material composition and nutritional levels of the forage are shown in Table 1. The Cd content of the ration was 0.06 mg/kg, which was in accordance with the Hygienical standards for feeds (GB 13078–2017). The temperature in the animal room was maintained at 30 ℃. After 10 d of feeding, the chickens were randomly divided into 4 groups with 3 replicates per group, and each replicate contained 4 chickens: control group, Cd group (70 mg/kg Cd) (Vromman et al., 2010), HNK group (200 mg/kg HNK) (Yuan et al., 2023), and Cd + HNK group (200 mg/kg HNK and 70 mg/kg Cd). Cadmium chloride hemi (pentahydrate) (CdCl_2_·5/2H_2_O, C805430, 98%) for animal experiments was purchased from Maclean Biochemical Technology Company (Shanghai, China), HNK (S31381) used for animal experiments was purchased from Yuanye Bio-Technology Company (Shanghai, China). During the animal experiment, the chickens were allowed to feed ad libitum and drink water freely. The wet feed was stirred with water, and HNK was added to the wet feed and mixed evenly. The chickens were continuously fed for 4 wk, and their body weight was measured and recorded every week. On the 29th d of the feeding period, counted chicken weights and liver weights, and the liver somatic index was analyzed according to the organ weight (g)/body weight (g) ratio. Blood samples were collected from the chickens and they were euthanized.

**Table 1:** The diet composition and nutritional composition of chicken basic diet.

Ingredients	Contents (%)	Nutrient components	Contents (%)
	1–13 wk old		1–13 wk old
Corn	60.42	CP≥	16.00
Soybean meal	33.00	EE≤	8.00
Soybean oil	2.60	Ash≤	15.00
CaHPO_4_	1.50	Ca	3.30–4.50
Limestone	1.40	TP≥	0.40
Premix	0.80	Nacl	0.30–0.80
*DL*-Met	0.16	Lys≥	0.85
Lys	0.12	Met	0.30–0.90
Total	100.00	Moisture ≤	14.00

The premix provides the following per kilogram of diet: VA 10 000 IU; VD_3_ 25 00 IU; VK_3_ 2.5 mg; VB_1_ 4 mg; VB_2_ 6 mg; Folic acid 100 mg; Pantothenic acid 120 mg; Niacin 50 mg; VB_12_ 0.5 mg; VE 30 mg; Biotin 0.15 mg; Cu 8 mg; Zn 80 mg; Fe 80 mg; Mn 100 mg; Se 0.45 mg; I 0. 40 mg.

### Serum Collection

The blood samples were centrifuged to obtain serum. A portion of liver tissue was fixed with tissue fixative and electron microscope fixative to prepare tissue and electron microscope sections, while another liver tissue was stored in liquid nitrogen for gene and protein level detection.

### Evaluation of Liver Indexes in Chickens

The chicken's blood samples were placed in 15 mL centrifuge tubes, and the blood samples were left to precipitate at 37 ℃ for 2 h. The samples were centrifuged to obtain the upper serum at 2,000 r/min on a low-speed centrifuge to obtain upper serum. alanine aminotransferase (**ALT**) and ALT activities were measured using the method described by Fan yang (Yang et al., 2019). Subsequently, serum samples from each group were used for the detection of ALT and aspartate aminotransferase (**AST**) levels using an automatic blood biochemical analyzer (Beckman AU480) at Yangzhou University Testing Center (Yangzhou, China).

Six liver tissue from each group were evenly ground on ice. Then, they were centrifuged at 2000 r/min in a 4 ℃ ultra-high-speed centrifuge and the supernatant was removed to perform MDA and for the determination of GSH content, CAT, GSH-Px, and T-SOD enzyme activity, all of these processes were performed on ice.

### Histopathological Evaluation

Hematoxylin and eosin staining was performed according to the procedures described by (Shi et al., 2020). The tissue was fixed with paraffin and the paraffin block section was dewaxed with PBS. The slices were stained with hematoxylin for 3 to 5 min, differentiated with aqueous hydrochloric acid solution, turned blue with aqueous ammonia solution, washed with water, dehydrated in a gradient of 85% and 95% alcohol, and stained with eosin staining solution for 5 min. Finally, the slices were dehydrated and mounted on slides. An upright microscope (OLYMPUS, BX53, Japan) was used to observe changes in tissue morphology and take pictures.

### Ultrastructural Analysis

Transmission electron microscopy (**TEM**) observations were made according to the procedure described by Xinrui Zhang (Zhang et al., 2023). The liver tissue was divided into 1 mm^3^ pieces and fixed in 2.5% glutaraldehyde at room temperature. This was followed by rinsing with 0.1 M phosphate buffer PBS (PH7.4) 3 times for 15 min each time. Multiple dehydration operations were performed sequentially using 50%, 70%, 80%, 90%, 95%, 100% ethanol, 100% acetone, and 100% acetone, each treatment time was 15 min. The treated samples were immersed in acetone:812 embedding agent=1:1 ratio solution for 2 to 4 h, then immersed in acetone: 812 embedding agent = 2:1 ratio solution overnight, and finally fixed in pure 812 embedding agent for 5 to 8 h, and then poured into the embedding plate and placed in the oven at 37℃ overnight. The treated samples were put into the oven at 60℃ for 48 h of continuous polymerization and embedding. Ultrathin sections were performed by slicing 60 to 80 nm with an ultrathin slicer. After gradient dehydration in ethanol and acetone, directional embedding was performed in epoxy resin; ultrathin sections were prepared and stained with uranyl acetate and lead citrate. All sections were observed under a transmission electron microscope (HT7800, Hita chi, Tokyo, Japan).

### TUNEL Staining

The staining was processed following the instruction of a TUNEL Apoptosis Detection Kit (Nanjing Jiancheng Institute of Bioengineering, China). The prepared paraffin block was dewaxed, and the membrane was ruptured with 0.1% Triton X-100, equilibrated at room temperature mixed with the appropriate amount of TDT enzyme, dUTP, and buffered in the TUNEL kit at a ratio of 1:5:50 according to the number of films and the size of the tissue. The slices were placed in a wet box and incubated for 2 h at 37 ℃, and a small amount of water was added to the wet box to maintain humidity. The nuclei were counterstained with 4′,6-diamidino-2-phenylindole (**DAPI**) and mounted. Pathological sections and TUNEL-stained sections were collected and observed under an upright microscope and an inverted fluorescence microscope, respectively.

### Quantitative Real-Time PCR

Liver tissues (50 mg) were taken from 4 groups of 6 different individuals, they were ground evenly with a grinding rod in ice PBS solution, and centrifuged at 2000 r/min for 10 min in a 4℃ high-speed centrifuge. Clean liver tissue was collected at the bottom of the centrifuge tube and dissolved in TRIzol solution (R0016, Beytime). The concentration and purity of total RNA were checked using a NanoDrop 2000/2000c spectrophotometer (USA). The purity of total RNA met the experimental requirement (OD260/OD280 between 1.8 and 2.0). cDNA was synthesized according to the manufacturer's instructions (Yeason, China). The obtained cDNA was diluted 10 times with sterile water and was stored at -80 ℃ for the next step. Real-time PCR (**qPCR**) with 20 μL of reaction mixture (Yeason, China) was performed using 7500 Real-Time PCR Instrument (96-well 0.2 mL Block) (Thermo Fisher Scientific). Finally, 2^−ΔΔCt^ method was used to calculate relative mRNA expression (Yuan et al.,2023). The primer sequences of genes are shown in Table 2, and all primers were synthesized by Huada Gene Biotech Co. Ltd (China).

**Table 2:** Primers used in experiments in this section.

Gene names	Primers sequence 5′→3′
*SIRT3* (CHICKEN)	F: CATCCCAGACTTCAGGTCTCC
	R: AGTGGGCGTAGTTGGGTCTA
*SOD2* (CHICKEN)	F: TGGGGGTGGCTTGGGTATAA
	R: CAGCAATGGAATGAGACCTGT
*β-actin* (CHICKEN)	F: CCGCTCTATGAAGGCTACGC
	R: CTCTCGGCTGTGGTGGTGAA
*HSP60* (CHICKEN)	F: AGCCAAAGGGCAGAAATG
	R: TACAGCAACAACCTGAAGA
*HSP70* (CHICKEN)	F: CGGGCAAGTTTGACCTAA
	R: TTGGCTCCCACCCTATCTCT
*HSP90* (CHICKEN)	F: TCCTGTCCTGGCTTTAGTTT
	R: AGGTGGCATCTCCTCGGT
*GPxl* (CHICKEN)	F: ACGGCGCATCTTCCAAAG
	R: TGTTCCCCCAACCATTTCTC
*GPx2* (CHICKEN)	F: ATCGCCAAGTCCTTCTACGA
	R: ACGTTCTCGATGAGGACCAC
*GPx4* (CHICKEN)	F: CTTCGTCTGCATCATCACCAA
	R: TCGACGAGCTGAGTGTAATTCAC
*IL-4* (CHICKEN)	F: AGGAAACCTCTCCCTGGATGTC
	R: AAATCCCTCCTCGCCAATCT
*IL-6* (CHICKEN)	F: CCCTCACGGTCTTCTCCATAAA
	R: TGTCTCACCTGCTATTTGCCTTAC
*TNF-γ* (CHICKEN)	F: CTGGAATCTCATGTCGTTCATCG
	R: ACGGCGCATCTTCCAAAG
*GJB1* (CHICKEN)	F: CAGTGGTGGACAGAGACGAG
	R: ATGGCTCCTCACGCCTGG

### Spectrophotometric Determination of Trace Elements

Trace elements were determined in digested solutions using Inductively Coupled Plasma-Optical Emission Spectrometry (Optima 2100, Perkin-Elmer, Shel ton, CT) (Osmond-McLeod et al., 2014). Liver tissue samples from each group were placed in different labeled 5 mL EP tubes and cut with scissors. The samples were kept at a temperature of 80℃ to dry out the water. The liver tissue was then ground into a powder. The tissue was digested using a microwave digestion instrument: 200 mg of liver powder and 4 mL of concentrated nitric acid were added into the digestion tubes, and the liver tissue was microwave-digested at 150℃ for 40 min. The digested tissue solution was then diluted with distilled water into a 10 mL solution and transferred to a new 15 mL centrifuge tube. The final elemental contents were analyzed using an atomic spectrometer.

### Statistical Analysis

Every experiment was repeated at least 3 times. All test results were calculated by GraphPad Prism software. One-way ANOVA was used to compare differences between groups. In comparison with the control group, *means a statistically significant difference (*P* < 0.05), and **indicates a highly significant difference (*P* < 0.01). In comparison with the Cd group, #means a statistically significant difference (*P* < 0.05), ##indicates a highly significant difference (*P* < 0.01).

## RESULTS

### Effects of HNK and Cd Feeding on Chicken Body Weight

**Figure 1:**
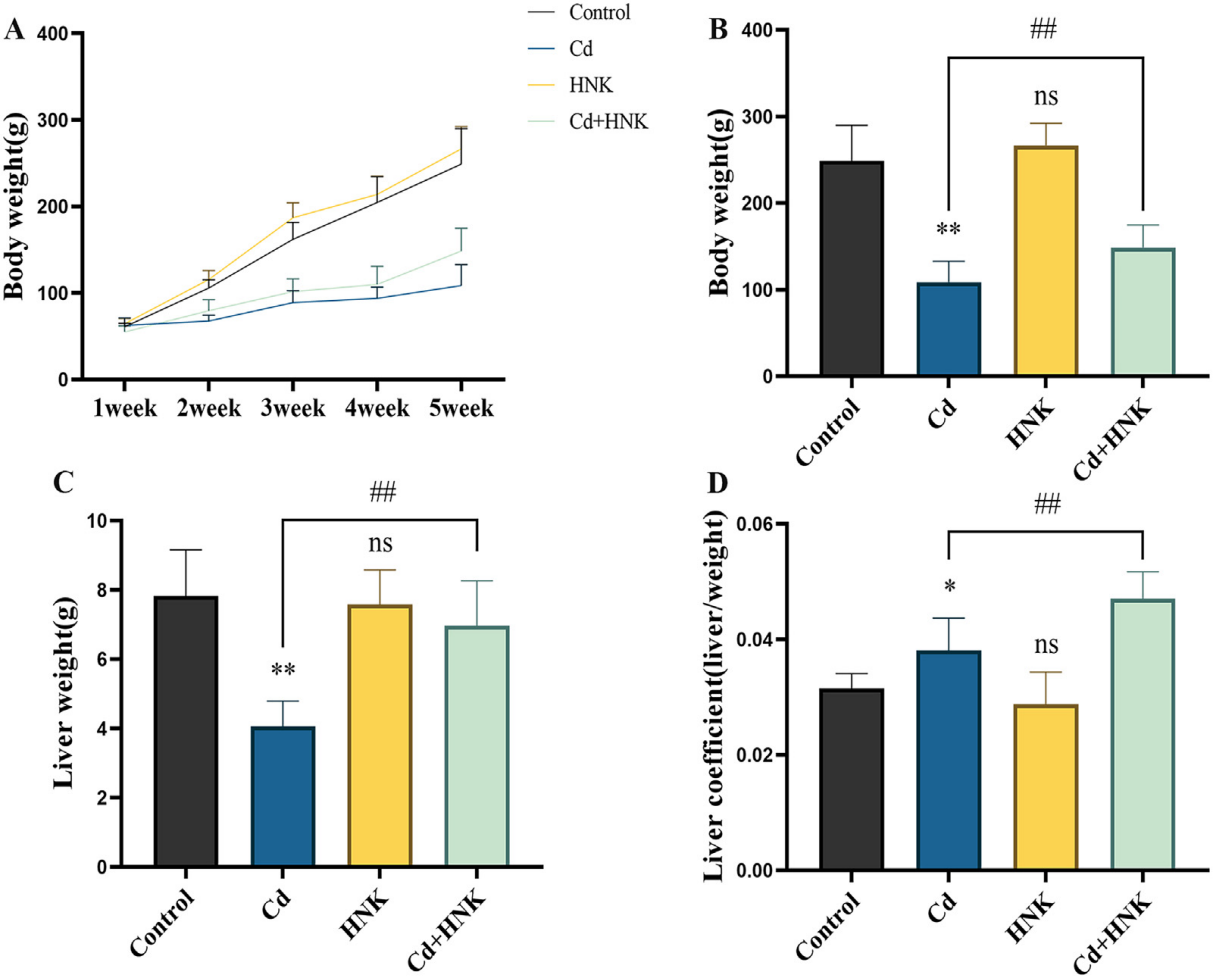
HNK mitigates Cd-induced impaired growth and development in chickens. (A) During the experiment, the weight of the chicken in each group was recorded on wk 1, 2, 3, 4, and 5. (B-D) After the experiment period, the final weight, liver weight, and liver coefficient of each group of chickens are counted and calculated. Note: N = 6, ns: *P*>0.05, compared with the control group, *: *P* < 0.05, **: *P* < 0.01; compared with the Cd group, #: *P* < 0.05, ##: *P* < 0.01.

The results showed slow increase in body weight in the Cd group and little difference in body weight in the HNK group compared to the control group. There was an increase in body weight in the Cd + HNK group compared to the Cd group (Figure 1A). the body weight and liver weight of chicken after the administration of Cd significantly reduced the both body and liver weight, while the liver coefficient was increased. Besides that, the Cd + HNK group shown a major improvement in above measured parameters compared to the Cd group (Figure 1B-D).

### Effect of HNK on Serum Markers of Cd-Induced Damage

**Figure 2:**
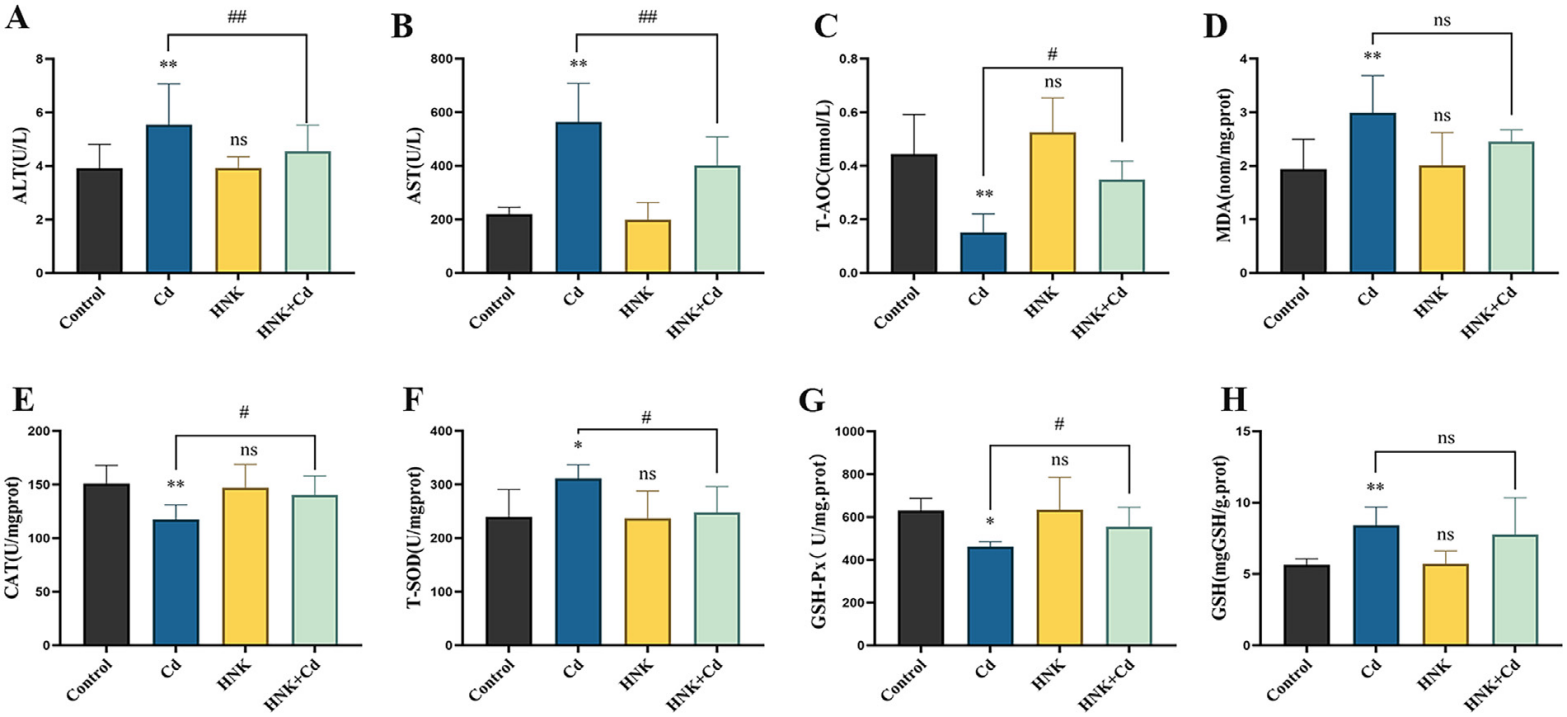
HNK alleviates Cd-induced serum index and antioxidant index disorders in chickens. (A-B) HNK improves the disturbance of cadmium on liver biochemical indexes ALT, and AST in chicken serum. (C-H) T-AOC content, MDA activity, GSH-Px activity, CAT activity, T-SOD activity, GSH content. Note: N = 6, ns: *P* > 0.05, compared with the control group, *: *P* < 0.05, **: *P* < 0.01; compared with the Cd group, #: *P* < 0.05, ##: *P* < 0.01.

The results showed that the levels of ALT and AST in the Cd group were higher than those in the control group (*P* < 0.01), differences in the HNK group were not significant (*P* > 0.01). Compare to the Cd group, the combination of HNK and Cd group improved the increase of liver indicators caused by Cd (*P* < 0.01) (Figure 2A-B).

### Effect of HNK on Antioxidant Capacity of Cd-Induced Damage in Chickens

The results showed that compared to the control group, Cd decreased the activity of T-AOC, CAT, and GSH-Px (*P* < 0.05 or *P* < 0.01), while increasing the levels of MDA and GSH (*P* < 0.01), also increased the activity of T-SOD (*P* < 0.05). The differences in the HNK group were not significant. However, compared to the Cd group, the Cd + HNK group alleviated the reduction in the activity of T-AOC, CAT, and GSH-Px caused by Cd, while relatively reducing the levels of MDA and GSH (*P*<0.01), and decreased the activity of T-SOD, which is not significant (*P* > 0.05) (Figure 2C-H).

### Effect of HNK on Histopathological Evaluation and Ultrastructural Analysis of Cd-Induced Damage in Chickens

**Figure 3:**
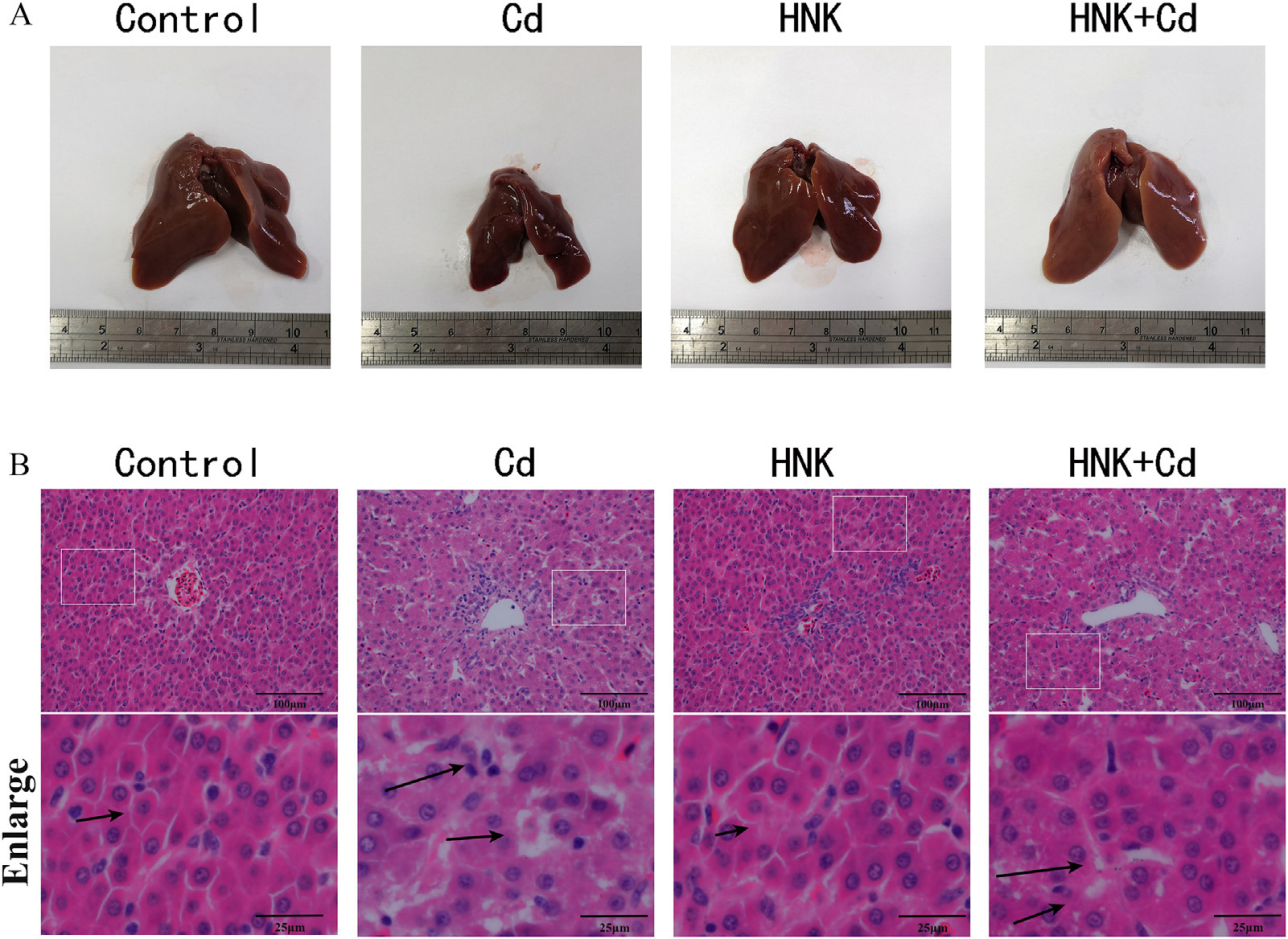
HNK protects chickens' liver tissue from Cd-induced pathological damage. (A) Macroscopical features of chicken liver. (B) Effects of HNK and Cd on chicken liver tissue observed by HE staining. Note: Arrows indicate hepatic cord.

**Figure 4:**
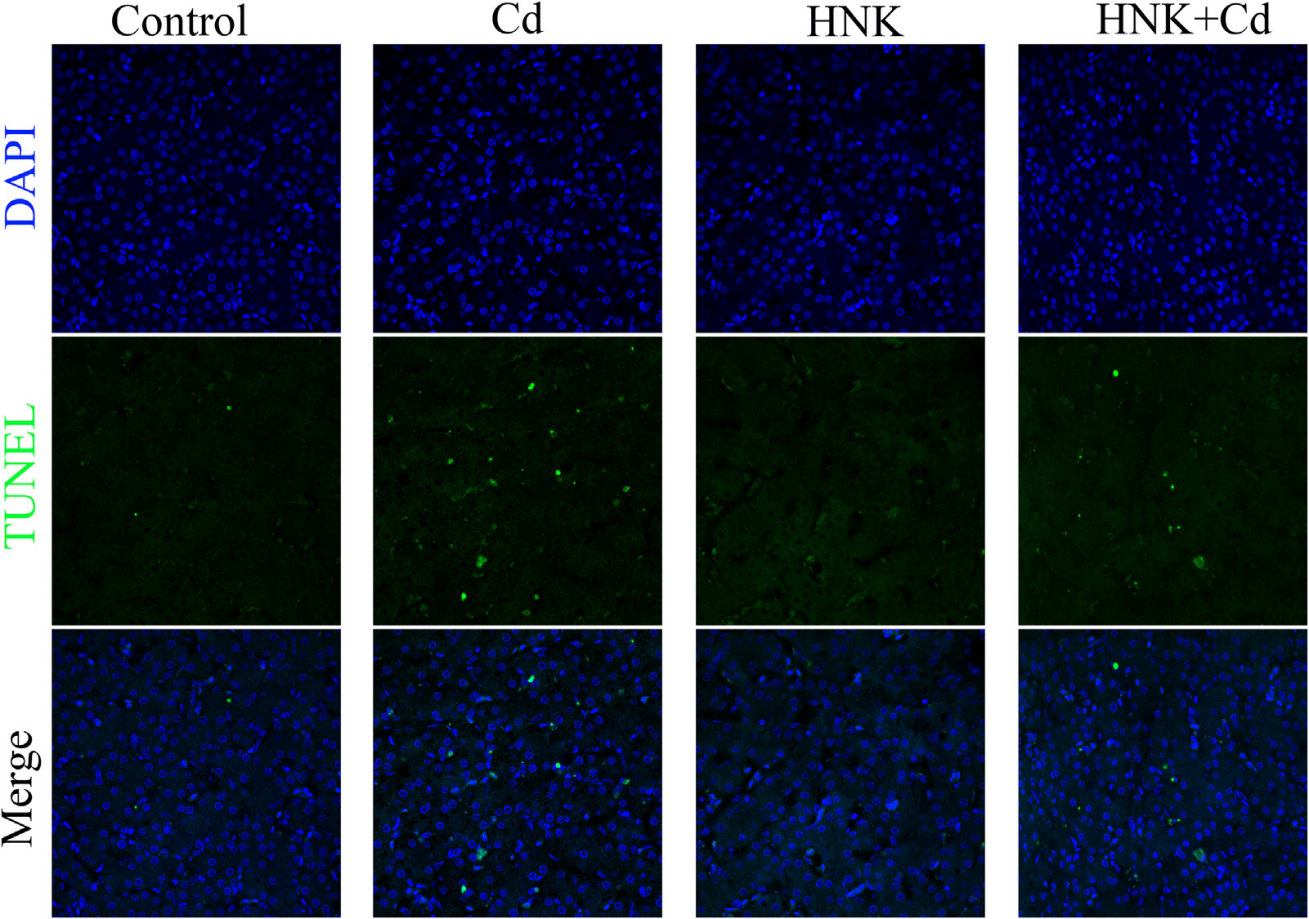
TUNEL staining of liver tissue with blue-labeled DAPI showing the location of nucleus. Scale bar = 100 μm.

Compared to the control group, the volume of the liver in the Cd group decreased, and the results of pathological sections showed that Cd caused the enlargement and disordered arrangement of hepatic cord space in chicken liver. Moreover, the volume and hepatic cord arrangement of the liver in the HNK group did not differ significantly from that of the control group. Compared to the Cd group, The liver cord disorder was improved in the Cd + HNK group (Figure 3A-B). In addition, the results showed that compared to the control group, a large number of TUNEL-positive sites were co-located with the nucleus in the Cd group, the HNK group exhibited no changes, while compared to the Cd group this phenomenon was improved in the Cd + HNK group, and the co-location of TUNEL-positive sites with the nucleus was reduced (Figure 4).

### The Contents of Trace Elements in Chicken Liver Tissue Under HNK Intervention

**Figure 5:**
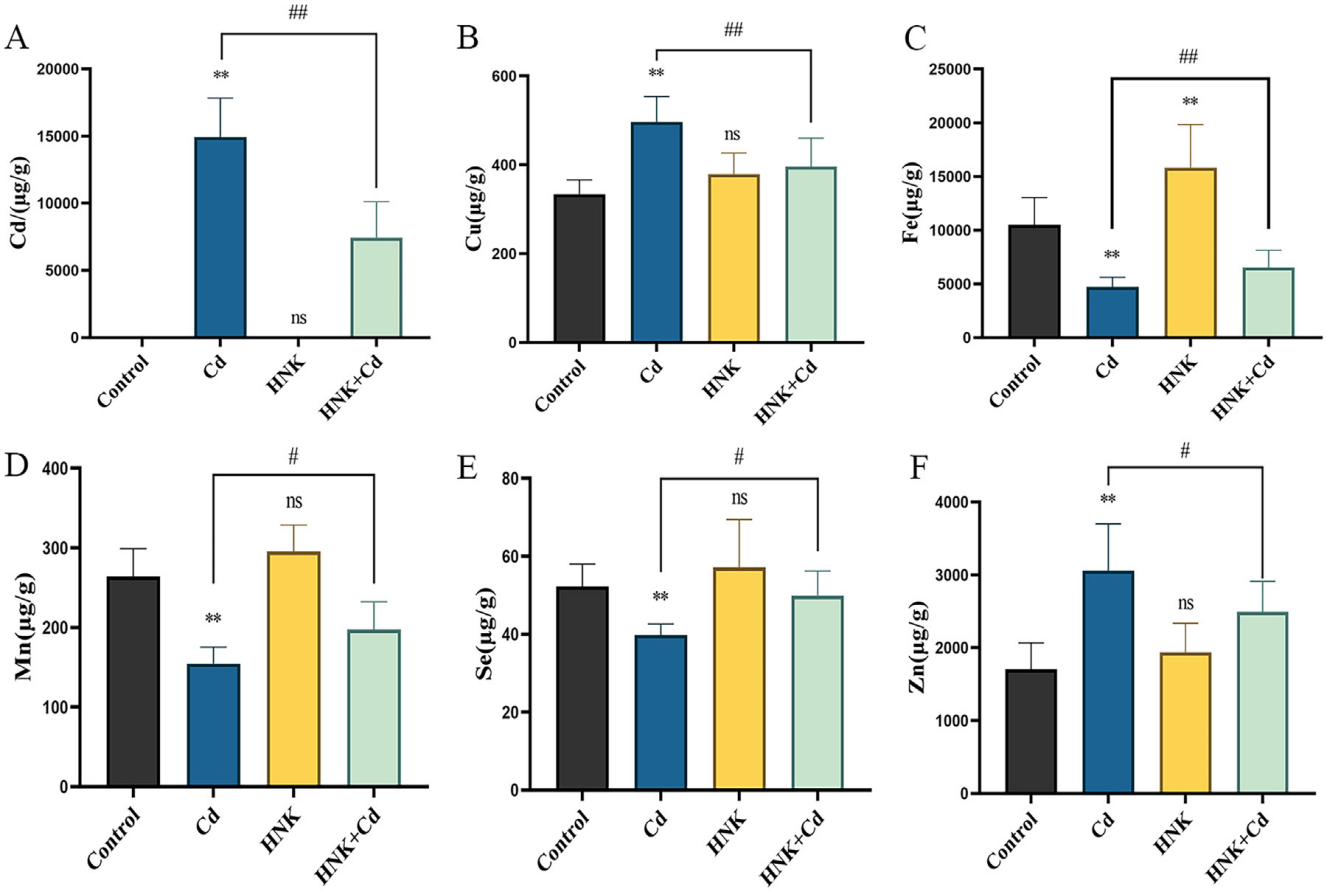
Microwave digestion and elemental analysis were performed to test the level of Cd and the 5 kinds of trace elements. (A) the level of Cd. (B) the level of Cu. (C) the level of Fe. (D) the level of Mn. (E) the level of Se. (F) the level of Zn. Note: N = 6, ns: *P* > 0.05, compared with the control group, *: *P* < 0.05, **: *P* < 0.01; compared with the Cd group, #: *P* < 0.05, ##: *P* < 0.01.

The results showed that compared to the control group, the Cd group exhibited an increase in Cd accumulation in the liver, and the HNK group did not lead to an increase in cadmium levels (*P* > 0.01), while the Cd + HNK group showed a noticeable decrease in Cd accumulation compared to the Cd group (*P* < 0.01). Compared to the Cd group, the Cd + HNK group showed a decrease in Cd content (*P* < 0.01) (Figure 5A). Compared to the control group, the levels of Fe, Mn and Se in the liver were decreased in the Cd group, while the levels of Cu and Zn were increased (*P* < 0.01). The HNK group caused a highly significant increase in Fe content (*P* < 0.01), but had little effect on Cu, Mn, Se, and Zn content (*P* > 0.01). Compared to the Cd group, the Cd + HNK group exhibited an increase in Fe, Mn and Se levels, while the levels of Cu and Zn were slightly reduced (*P* < 0.01 or *P* < 0.05) (Figure 5 B-F).

### Effect of HNK on the Expression of Cytokines and Antioxidant Proteins of Cd-Induced Damage in Chickens

**Figure 6:**
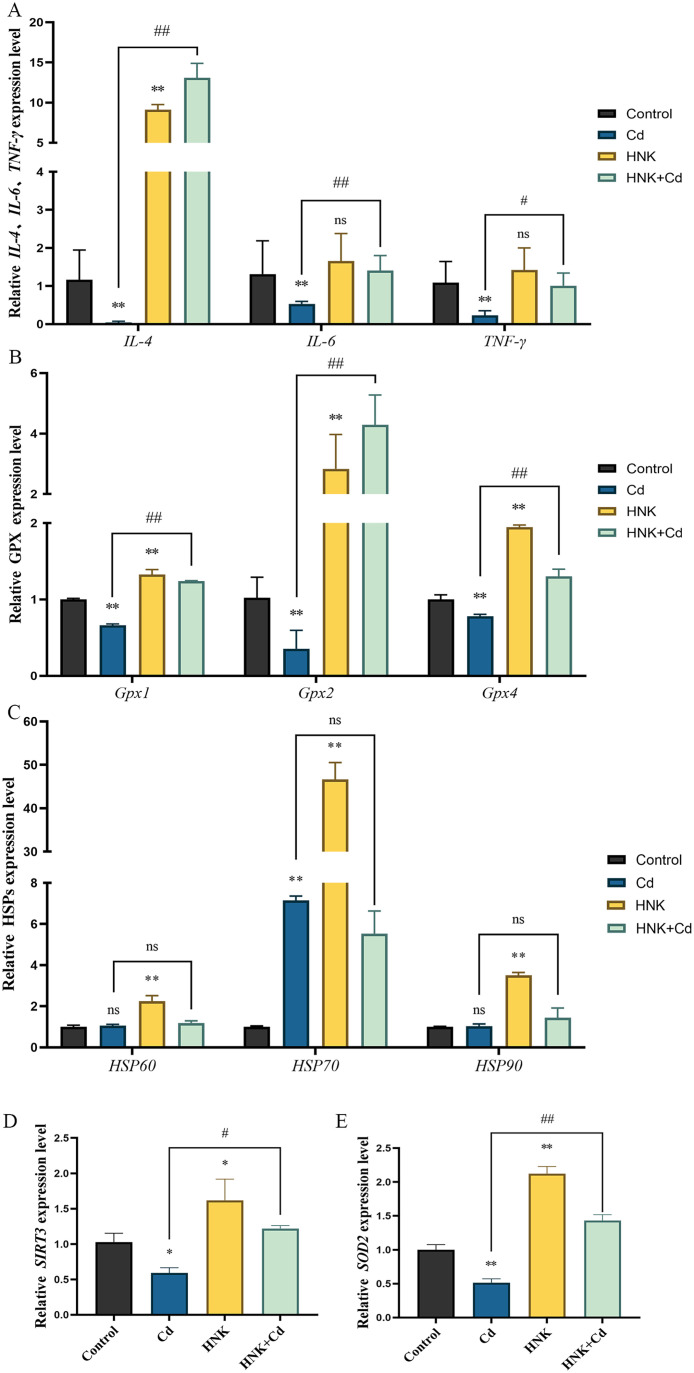
Detection of the gene expression levels by qPCR. (A) mRNA levels of *IL-4,IL-6,TNF-γ.*(B) mRNA levels of *Gpx1,Gpx2,Gpx4*. (C) mRNA levels of *HSP60,HSP70,HSP90*. (D) mRNA levels of *SIRT3*. (E) mRNA levels of *SOD2*. Note: N = 6, ns: *P* > 0.05, compared with the control group, *: *P* < 0.05, **: *P* < 0.01; compared with the Cd group, #: *P* < 0.05, ##: *P* < 0.01.

The results showed that compared with the control group, the expression levels of several cytokines such as *IL-4, IL-6*, and *IFN-γ* were decreased (*P* < 0.01), the gene expression levels of antioxidant genes *Gpx1, Gpx2*, and *Gpx4* were decreased in the Cd group (*P* < 0.01), the expression levels of *HSP70* were significantly increased in the Cd group (*P* < 0.01), but the expression levels of *HSP60* and *HSP90* were not significantly different (*P* > 0.05). And the expression levels of *HSP60, HSP70* and *HSP90* in HNK group were significantly increased (*P* < 0.01). The expression levels of HNK group causes a significant increase in the expression levels of the genes *IL-4, Gpx1, Gpx2*, and *Gpx4*, but has no significant effect on the expression levels of the other genes (*P* > 0.05). Compared with the Cd group, the expression levels of several cytokines such as *IL-4, IL-6, and IFN-γ* were also increased in the Cd + HNK group, and the levels of antioxidant genes *Gpx1, Gpx2*, and *Gpx4* were also increased (*P* < 0.01), but has no significant effect on the expression levels of the *HSP60, HSP70, HSP90* (*P* > 0.05) (Figure 6).

### Effect of HNK on SIRT3-SOD2 Pathway of Cd-Induced Damage in Chickens

Compared with the control group, the mRNA expression of *SIRT3* and *SOD2* were decreased in the Cd group, while the mRNA expression of *SIRT3* and *SOD2* were increased in the HNK group (*P* > 0.05 or *P* < 0.01). Compared with the Cd group, the levels of *SIRT3* and *SOD2* were recovered in the Cd +HNK group (Figure 6D-E). These results indicated that HNK improved the damage of *SIRT3* and *SOD2* horizontal pathways of Cd mitochondria and reduced the damage of mitochondria.

**Figure 7:**
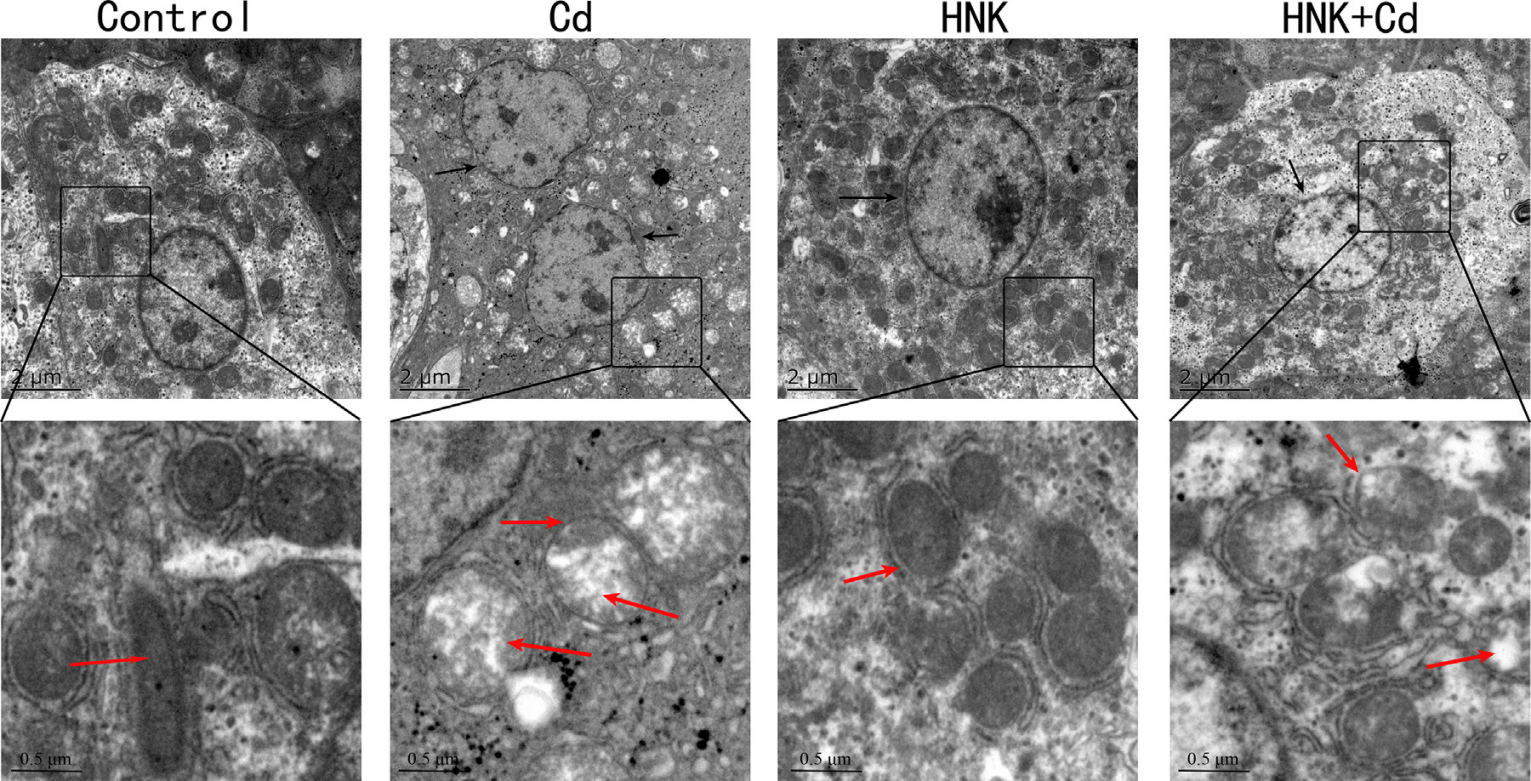
Effects of Cd and HNK on liver ultrastructure in chickens. Observation of Cd-induced ultrastructure damage of liver cells by transmission electron microscopy. Note: The black arrows indicate the nuclear membrane of the nucleus and the red arrows indicate the mitochondrial cristae.

By observing the ultrastructure of chicken liver tissue, it was found that compared with the control group, the Cd group could cause nuclear membrane dissolution, mitochondrial vacuolization, and mitochondrial ridge disappearance. There was no significant difference between the HNK group and the control group. Compared with the Cd group, the damage of nuclear membrane and mitochondria in the Cd + HNK group was alleviated (Figure 7C).

### Effect of HNK on Gap Junction Protein of Cd-Induced Damage in Chickens

**Figure 8:**
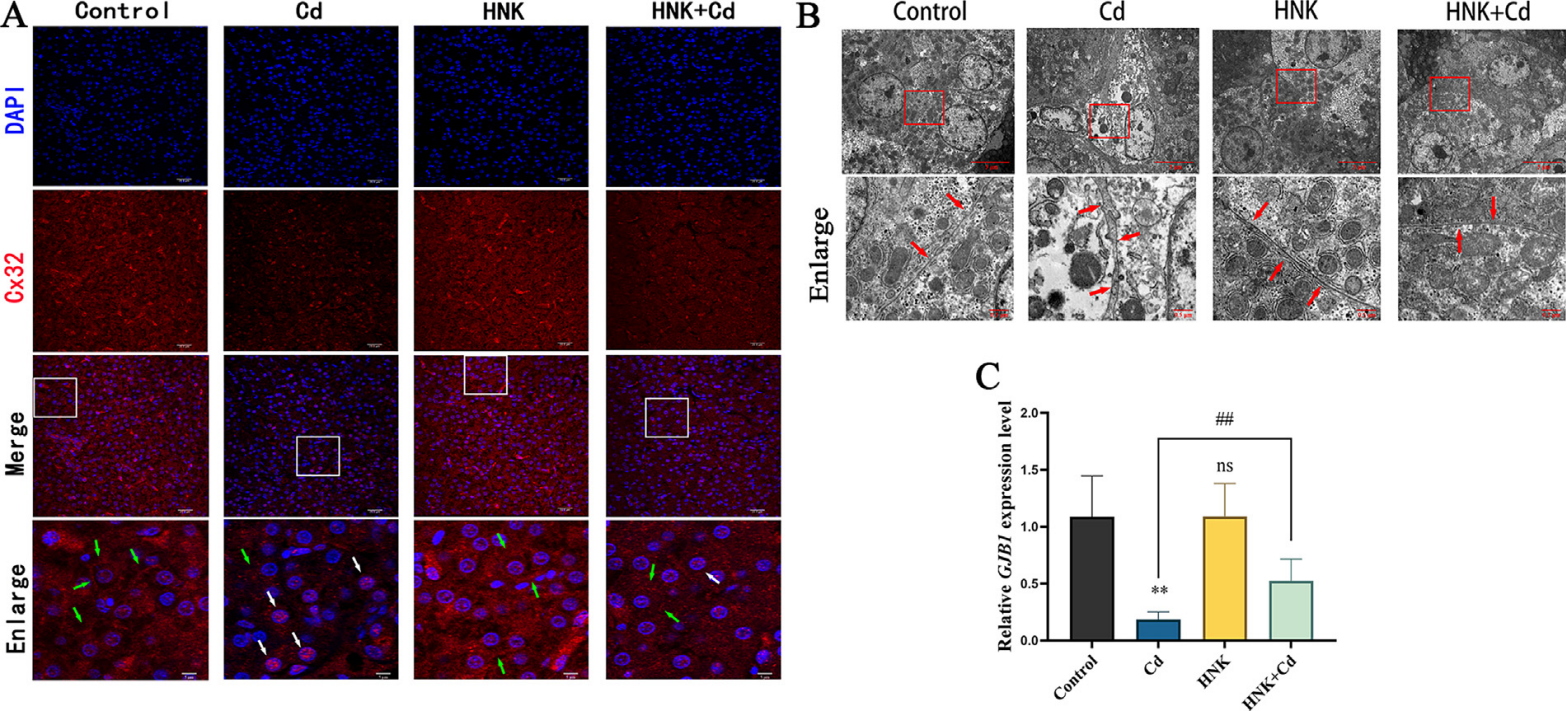
(A) Effects of combined treatment of Cd and HNK on Cx32 distribution. Scale Bar = 10 μm. Observation of Cd-induced ultrastructure damage of liver cells by transmission electron microscopy. (B) Observation of Cd-induced ultrastructure damage of liver cells by transmission electron microscopy. (C) mRNA levels of *GJB1*. Note: N = 6, ns: *P* > 0.05, compared with the control group, **: *P* < 0.01; compared with the Cd group, ##: *P* < 0.01. The green arrow represents the gap junction and the white arrow represents gap junction protein near nucleus, the red arrow represents the connection between cells.

The distribution of Cx32 in the gap junction protein was uniform in both the control group and the HNK group, whereas Cx32 accumulated in the nucleus in the Cd group. The accumulation of Cx32 protein was alleviated in the Cd + HNK group (Figure 8A). Transmission electron microscopy revealed that the structure of gap junctions appeared intact in the control group and the HNK group, while they were disordered in the Cd group, this disorder was relieved in the Cd + HNK group (Figure 8B). Additionally, compared to the Control group, there was a significant decrease observed in *GJB1* gene transcription level with the Cd group (*P* < 0.01), with no notable difference between the HNK group and the control group (*P* > 0.05). Furthermore, relative to the Cd group, there was a decrease in *GJB1* gene transcription level for the Cd + HNK group (*P* < 0.01) (Figure 8C).

## DISCUSSION

Cadmium (**Cd**) pollution is an environmental problem that has existed for a long time and is getting more and more serious (Zhu et al., 2020). Cd in the environment is absorbed by plants and eventually by livestock and poultry, which will greatly affect the growth and development of livestock and poultry, and ultimately affect people's health. HNK, a new type of natural lignan plant extract, which is widely used in various aspects of food (Sarrica et al., 2018). We found that adding HNK to the diet increased the body weight of chickens, demonstrating its growth-promoting effects. The liver, being the largest metabolic and detoxification organ in animals, is significantly affected by Cd. When Cd enters the chicken's digestive system, it is absorbed and gradually accumulates in various organs and tissues via the bloodstream. In the liver, Cd depletes antioxidant substances, causing liver cells to weaken and die, thereby impairing liver metabolism and protein synthesis. This disruption leads to weight loss and hampers normal development. HNK alleviated Cd-induced liver weight loss. Notably, the liver weight in the Cd + HNK group was higher than in both the control and HNK group, likely due to excessive compensatory mechanisms. Visually, livers from the Cd group were smaller, whereas the Cd + HNK group showed improvements in liver weight and size. Pathological examination revealed that Cd exposure led to the disappearance of hepatic cords, disorganized hepatocyte arrangement, increased intercellular space, and hepatic sinusoid atrophy and deformation. In contrast, the Cd + HNK group showed improved hepatic cord organization, more compact hepatocyte arrangement, and better cell-to-cell contact. Additionally, HNK reduced the serum levels of ALT, AST, and LDH, indicating a protective effect against Cd-induced liver damage. (Jeong et al., 2018) also showed that HNK could reduce markers of liver injury. By TUNEL staining, we found that HNK alleviated Cd-induced colocalization of TUNEL dye to the nucleus compared with the Cd group, suggesting that HNK alleviated Cd-induced apoptosis of hepatocytes. It is indicated that HNK has protective effect on liver injury caused by Cd. Some studies have also shown that HNK can improve liver dysfunction in mice (Lee et al., 2015). These results are consistent with the results of our experiment.

The activities of CAT, GSH-Px, and T-SOD in the liver are an important reflection of the level of liver antioxidant function. GSH is an important antioxidant substance in cells. Studies have shown that Cd efflux is dependent on GSH (Sandbichler and Höckner, 2016). MDA is an oxidative stress factor produced by heavy metal damage to cell structure. Our results showed that HNK mitigated the Cd-induced decrease in the activities of CAT, GSH-PX, and T-SOD, and decreased the Cd-induced increase in the levels of GSH and MDA. This is consistent with the results of Xiaolei Miao (Miao et al., 2023). As a member of the iron/manganese superoxide dismutase family, SOD2 can clear ROS and has a protective effect on cell death (Qiu et al., 2010). Sirtuin3 (**SIRT3**) is an NAD^+^-dependent deacetylase that regulates mitochondrial oxidative homeostasis by deacetylating a variety of substrates (Ansari et al., 2017). The interaction between SIRT3 and HNK may enhance the deacetylase activity of SIRT3 (Pillai et al., 2015). It has been shown that HNK can enhance mtROS clearance by increasing SIRT3-mediated SOD2 activity and transcription under fluoride exposure (Wang et al., 2022). Huifeng Pi's study shows that SIRT3-SOD2 mediated autophagy is an important mechanism of Cd-induced liver toxicity (Pi et al., 2015). In this study, the levels of *SIRT3* and *SOD2* in the liver of chickens in the HNK group were slightly increased, and HNK could ameliorate the Cd-induced reduction of *SIRT3* and *SOD2* mRNA transcription levels, which was consistent with the above results. Earlier studies in our laboratory also confirmed that HNK can activate the SIRT3-SOD2 pathway to alleviate Cd-induced cell damage in BRL 3A cells (Yuan et al., 2022).

Cd can also promote oxidative damage by disrupting the homeostasis of trace elements (Ge et al., 2019). Trace elements are essential cofactors with critical roles in cellular antioxidant mechanisms and metabolism. Cd in the environment often enters animal cells by competing with essential metal ions for binding to functional proteins, leading to metabolic disorders and trace element depletion. Studies have shown that Cd can displace iron, manganese, and other trace elements, reducing their levels in the body (Choong et al., 2014). Our experiment found that short-term acute Cd poisoning had different effects on the contents of trace elements in the liver of chickens. Cd caused a decrease in the contents of iron and selenium but increased the contents of zinc, manganese, and copper. This result is consistent with our results in quail, and HNK greatly reduces Cd accumulation in the liver, which we hypothesize is because HNK improves the overall capacity of liver cells to expel Cd. Zinc has been shown to promote the synthesis and gene expression of metallothionein, which can mitigate its toxic effects by binding to Cd. Copper and zinc as cofactors of endogenous antioxidant enzyme Cu-Zn superoxide dismutase can express antioxidant effect by scavenging superoxide anion free radicals (Bhattacharya, 2022). We also found that single HNK added to the feed generally can make chicken liver of iron, copper, zinc, manganese, and selenium content increased, due to the importance of trace elements in animal growth and development, this explains HNK may be due to the reason for the chicken body weight increase, HNK improved the level of trace elements in liver tissue. The addition of HNK to the diet can alleviate the metabolic disorder of trace elements in chicken liver caused by Cd, alleviate the accumulation of Cd in chicken liver, and improve the weakening of antioxidant capacity and the decline of antioxidant gene expression in the liver.

Glutathione peroxidase family (**GPX**), as one of the antioxidant enzyme family, acts synergistically with superoxide dismutase and catalase to form an enzymatic antioxidant system. GPX1 generally exists in the cytoplasm and mitochondria, catalytic glutathione reduces the toxicity of peroxide in the body. GPX2 is mainly expressed in the gastrointestinal system, but can also be detected in the liver. GPX4 is known as a lipid peroxidation inhibitor protein, and its antioxidant effect is closely related to ferrous iron (Jia et al., 2020). Research has shown that when mice selenium content is reduced, *GPX1* mRNA expression quantity will be less, consistent with the experimental results. In addition, the mRNA expression levels of *GPX2* and *GPX4* also decreased in this experiment, suggesting that Cd could induce oxidative stress injury in the liver, after HNK intervention, the above indicators were reversed and the oxidative stress injury in the liver was relieved, indicating that HNK could play a protective role in Cd-induced liver injury.

In addition, heat shock proteins (**HSPs**) are intracellular proteins that are synthesized to resist the damage of toxicants when cells are exposed to a variety of hazards. According to the features of HSPs generation can be speculated that Cd to various kinds of organelles in the cell damage, leading to a large number of abnormal proteins Hsp40 has been proven and Hsp70, a total of positioning, and as a common partner of Hsp70 to function. The primary function of Hsp40 is to regulate ATP-dependent peptide binding through Hsp70 (Kim et al., 2008). Experiments in a mouse model in which diabetes accelerates atherosclerosis have shown that *Hsp90* is associated with inflammation and oxidative stress (Lazaro et al., 2015). The results of this experiment show that Cd induced up-regulation of *HSP70*, but no affected the gene expression of *HSP60* and *HSP90*. However, the HNK protective the body by up-regulating the gene expression of *HSP*s. On the contrary, Cd inhibited the expression of cytokines in the liver tissue at the gene level, suggesting that Cd weakened the liver's immune level because these cytokines are not only involved in inflammation and other processes but also play a crucial role in the immune activity of liver cells. In this study, Cd decreased the levels of inflammation-related factors *IL-4, IL-6*, and *IFN-γ*. These *IL-4, IL-6*, and *IFN-γ* secreted by cells have also been shown to play a role in oxidative stress-induced inflammation (Arulselvan et al., 2016). Byung Hun KIM (Kim and Cho, 2008) has shown that HNK achieves anti-inflammatory effects by inhibiting the PI3K/Akt pathway. In this study, HNK can enhance the mRNA expression level of *IL-4, IL-6, and IFN-γ*, indicating that HNK has a certain anti-inflammatory effect.

Gap junction intercellular communication (**GJIC**) is a way for multicellular organisms to maintain cell homeostasis. The main connexins of the liver (hepatocytes) are connexin 32 (Cx32) and connexin 26 (Cx26) (García et al., 2016). It was shown that Cd could cause a decrease in the expression of *GJB1* in mouse liver (Jeong et al., 2000). Gap junctions are anchored to the cell membrane by microfilaments and microtubules (Goodson and Jonasson, 2018). Earlier studies by our group found that Cd disrupted the microtubule structure of BRL 3A cells and caused a decrease in *GJB1* expression. The tube was fixed to the cell membrane (Yuan et al., 2022). In this experiment, the results of tissue immunofluorescence staining showed that the distribution of Cx32 protein was absent at the cell boundaries of the Cd group compared with the control group, and Cx32 protein was aggregated towards the nucleus, indicating that Cx32 protein was internalized and degraded (Yuan et al., 2022). Compared with the Cd group, the distribution of Cx32 between cells in the Cd + HNK group was partially restored. The protective effect of HNK on Cx32 might be attributed to its resistance to Cd-induced oxidative stress. The results of transmission electron microscopy also further indicated that Cd could cause damage to the tissue cell junction, while HNK could alleviate the damage.

## CONCLUSIONS

In conclusion, HNK can alleviate the liver dysfunction caused by Cd in chicken liver, relieve liver injury through oxidation reaction and inflammation, and restore the gap junction between liver cells damaged by Cd. These results suggest that HNK can be used as an antidote to relieve liver toxicity caused by Cd in chickens.

## DISCLOSURES

The authors declare that they have no known competing financial interests or personal relationships that could have appeared to influence the work reported in this paper.
